# Resting-State Functional Connectivity between Fronto-Parietal and Default Mode Networks in Obsessive-Compulsive Disorder

**DOI:** 10.1371/journal.pone.0036356

**Published:** 2012-05-03

**Authors:** Emily R. Stern, Kate D. Fitzgerald, Robert C. Welsh, James L. Abelson, Stephan F. Taylor

**Affiliations:** 1 Departments of Psychiatry and Neuroscience, Mount Sinai School of Medicine, New York, New York, United States of America; 2 Department of Psychiatry, University of Michigan, Ann Arbor, Michigan, United States of America; 3 Department of Radiology, University of Michigan, Ann Arbor, Michigan, United States of America; Bellvitge Biomedical Research Institute-IDIBELL, Spain

## Abstract

**Background:**

Obsessive-compulsive disorder (OCD) is characterized by an excessive focus on upsetting or disturbing thoughts, feelings, and images that are internally-generated. Internally-focused thought processes are subserved by the “default mode network" (DMN), which has been found to be hyperactive in OCD during cognitive tasks. In healthy individuals, disengagement from internally-focused thought processes may rely on interactions between DMN and a fronto-parietal network (FPN) associated with external attention and task execution. Altered connectivity between FPN and DMN may contribute to the dysfunctional behavior and brain activity found in OCD.

**Methods:**

The current study examined interactions between FPN and DMN during rest in 30 patients with OCD (17 unmedicated) and 32 control subjects (17 unmedicated). Timecourses from seven fronto-parietal seeds were correlated across the whole brain and compared between groups.

**Results:**

OCD patients exhibited altered connectivity between FPN seeds (primarily anterior insula) and several regions of DMN including posterior cingulate cortex, medial frontal cortex, posterior inferior parietal lobule, and parahippocampus. These differences were driven largely by a reduction of negative correlations among patients compared to controls. Patients also showed greater positive connectivity between FPN and regions outside DMN, including thalamus, lateral frontal cortex, and somatosensory/motor regions.

**Conclusions:**

OCD is associated with abnormal intrinsic functional connectivity between large-scale brain networks. Alteration of interactions between FPN and DMN at rest may contribute to aspects of the OCD phenotype, such as patients' inability to disengage from internally-generated scenarios and thoughts when performing everyday tasks requiring external attention.

## Introduction

Obsessive-compulsive disorder (OCD) is characterized by intrusive thoughts, feelings, or images (obsessions) and repetitive behaviors (compulsions) aimed at reducing anxiety associated with obsessions. Neuroimaging studies examining brain activation in OCD at rest, during symptom provocation, and in response to cognitive tasks have made critical advances in elucidating the neurobiological substrates of the disorder, pointing to dysfunction in several cortical and subcortical regions. While dysfunction in orbital, medial frontal, and striatal areas composing fronto-striatal circuits [1] has long been found to be contribute to the pathogenesis of OCD [2]–[7], emerging evidence suggests there is broader cortical dysfunction in the disorder, including abnormality of dorsolateral prefrontal cortex, anterior insula, lateral and medial temporal lobe regions, and parietal cortex [8]–[15]. Such widespread alterations may be due to the diversity of tasks used to investigate OCD, which include response inhibition, habit formation, set switching/reversal, and performance monitoring, to name just a few. Contributing to the complexity of interpretation is the fact that the direction of group effects in the majority of these brain regions appears to differ depending on the task being used. The investigation of large-scale functional networks during rest in OCD has the advantage of identifying neural mechanisms that are not specific to the task employed, which will complement and extend findings from task-based studies.

A growing body of literature in neuroscience has begun to emphasize the importance of interactions between brain regions, due to the realization that a typical brain area is likely to support multiple cognitive functions and that unique functionality is most likely to emerge through inter-regional connectivity [16], [17]. Resting-state functional connectivity analysis using functional magnetic resonance imaging (rs-fcMRI) examines correlations between low-frequency bold fluctuations (LFBFs) at rest, allowing for the identification of regions or systems showing agonistic interactions (identified by positive correlations) as well as antagonistic or competitive interactions (identified by negative correlations). Numerous rs-fcMRI studies have identified “intrinsic" large-scale brain networks that exhibit interactions at rest similar to those identified during task [18]–[21]. Despite being task-independent, resting-state network connectivity is modulated by the preceding task [22], [23] and is related to task-evoked neural activity and behavior [24], [25], but with considerably more stability over time than task-related BOLD changes [26], [27]. Evidence suggests that changes in rs-fcMRI reflect altered network functioning in several disease states [28], indicating that this marker of brain function can provide important information about neurocircuit abnormalities in psychiatric disorders.

Recent investigations of rs-fcMRI in OCD focusing on the striatum [29]–[31] have supported fronto-striatal theories by identifying altered functional coupling between striatum and multiple regions of frontal cortex. Zhang et al [32] found altered resting-state connectivity within a “top-down control network" including posterior temporal cortex, lateral frontal and cingulate cortex, and precuneus, regions that partially overlap with the fairly well-delineated fronto-parietal network (FPN) composed of anterior insula, medial frontal cortex, and lateral frontal and parietal regions [33]. Although there are dissociable nodes within FPN [20], [34], as a whole this network is referred to as “task-positive" because it increases in activity when attention is directed to external stimuli in cognitive tasks [20], [34]–[36]. By contrast, the “default mode" network (DMN), composed of midline frontal and parietal areas, posterior inferior parietal lobule, and medial and lateral temporal lobe regions, often decreases in activity when attention is directed externally [18], [37], [38], but increases in response to a variety of introspectively-oriented cognitive processes including autobiographical memory, imagination, and thinking about the self [39], [40]. While the relationship between FPN and DMN during task depends upon the paradigm employed and the goals of the subject [41], [42], reductions in DMN activity [43], [44] and greater negative correlations between FPN and DMN [45] are associated with improved performance (and less “mind wandering") on tasks requiring externally-directed attention. FPN and DMN are also negatively correlated at rest, at least in healthy controls [18], [35], [46], suggesting that the unconstrained brain may be intrinsically organized to support competitive relationships between networks involved in external attention and internally-focused thought processes.

The investigation of competitive interactions between FPN and DMN at rest is particularly relevant for the study of OCD. Not only are nodes of these networks found to be abnormal during task-based studies of OCD, but the phenomenology of the disorder is consistent with the idea of a disrupted relationship between ongoing internal thought and external information, in that patients often excessively focus on internally-generated fears that are inconsistent with evidence present in the external environment [47]. To test the possibility that OCD patients show alterations of the intrinsic functional relationship between fronto-parietal and default mode networks, the current study measured whole-brain rs-fcMRI in OCD patients and control subjects using seeds located in fronto-parietal network. We predicted that negative correlations between FPN and DMN would be evident in controls, replicating prior studies using rs-fcMRI, but reduced or absent in patients with OCD.

## Materials and Methods

### Subjects

This research was approved by the Institutional Review Board of the University of Michigan Medical School, following the principles set forth by the Declaration of Helsinki. All subjects provided written informed consent. Resting-state functional connectivity data were acquired for a total of 69 subjects. Seven subjects were excluded due to technical problems (failure during data acquisition: 1, corrupted data: 4, poor coregistration: 2), leaving a total of 62 participants including 30 OCD patients and 32 control subjects for further analysis. Seventeen OCD patients were unmedicated for a minimum of 6 months prior to study participation (uOCD) and 13 were medicated (mOCD), primarily with serotonin-reuptake inhibitors (SRIs, see Table S1). All patients met DSM-IV criteria for current OCD, excluding primary hoarding subtypes. Due to the high comorbidity between OCD and depression [48], we did not want to bias our sample by excluding patients with histories of depression or sub-threshold depressive symptoms. Thus, prior history of major depression was allowed if it was in partial or full remission (n = 14), as was depressive disorder not otherwise specified (NOS) and dysthymia (n = 6). Importantly, no subjects were in a major depressive episode. Other axis I disorders were also excluded unless they were clearly secondary to the diagnosis of OCD, which were relatively few in number (specific phobia: n = 5, panic disorder NOS: n = 1; panic disorder: n = 2; eating disorder NOS: n = 2; anorexia nervosa, n = 3, trichotillomania: n = 1; chronic motor tic disorder, n = 1; bipolar disorder NOS: n = 1).

The control group included 17 unmedicated healthy control subjects (uHC) without psychiatric diagnoses and 15 medicated patient controls (mPC). Subjects with any history of OCD were excluded from both control groups. Subjects in the mPC group were patients with remitted major depression who were on SRI medication (Table S1) and had few comorbidities (panic disorder: n = 1; attention deficit hyperactivity disorder: n = 1; anxiety disorder NOS: n = 2; impulse control disorder NOS: n = 1). As many OCD patients were taking SRIs and had histories of depression, the current design allowed us to better localize group differences to the presence of OCD by comparing the OCD group with a control group also containing medicated participants with histories of depression.

Subjects were evaluated by a trained clinician using the Structured Clinical Interview for DSM-IV (SCID; [49]. Symptoms of anxiety and depression were quantified using Hamilton Ratings Scales for Anxiety (HAM-A; [50] and Depression (HAM-D; [51]. Obsessive-compulsive (OC) symptom severity (current and lifetime) was quantified using the Yale-Brown Obsessive-Compulsive Scale (Y-BOCS, [52]. Table 1 shows demographic and clinical information for the groups. Both OCD groups showed significantly more generalized anxiety and depression than either control group, as would be expected, but mOCD and uOCD patients were not different from each other. While OCD patients were not significantly different in age from the control group, they had attained fewer years of education at the time of testing.

**Table 1:** Demographic information.

	uOCD (n = 17)		mOCD (n = 13)		uHC (n = 17)		mPC (n = 15)		Group differences	Post-hoc comparisons
	*mean*	*sd*	*mean*	*sd*	*mean*	*sd*	*mean*	*sd*		
Age	23.0	5.2	28.6	8.2	25.4	7.7	31.3	9.3	**M**: F (1,58) = 8.7	mOCD>uOCD
									p = .005	mPC>uOCD
										all p<.05
Education (years)	14.8	1.3	15.7	2.8	15.9	2.4	17.0	2.0	**D**: F(1,58) = 5.0	mPC>uOCD
									p = .023	p<.01
HAM-A	8.8	4.0	8.1	4.1	1.4	1.7	4.5	2.6	**D:** F (1,58) = 45.3	uOCD, mOCD>uHC
									p<.001	uOCD, mOCD>mPC
									**D X M:** F (1,68) = 5.3	mPC>uHC
									p = .025	all p<.05
HAM-D	8.6	2.7	7.1	3.2	1.3	1.5	4.2	2.7	**D:** F (1,58) = 60.8	uOCD, mOCD>uHC
									p<.001	uOCD, mOCD>mPC
									**D X M:** F (1,68) = 11.8	mPC>uHC
									p = .001	all p<.05
Gender	9F/8M		6F/7M		9F/8M		8F/7M		ns	
YBOCS	23.4	3.7	20.4	5.1						

uOCD = unmedicated OCD, mOCD = medicated OCD, uHC = unmedicated healthy controls, mPC = medicated patient controls. Group differences in age, years of education, Hamilton Anxiety Scale score (HAM-A), and Hamilton Depression Scale score (HAM-D) were evaluated with separate 2×2 ANOVAs using diagnosis (OCD, controls) and medication (unmedicated, medicated) as between-subjects factors. Chi-square tests compared gender (all groups), while independent samples t-tests were used to compare mOCD and uOCD groups on Yale-Brown Obsessive Compulsive Scale (Y-BOCS) scores. sd = standard deviation, D = main effect of diagnosis factor, M = main effect of medication factor, D×M = interaction between diagnosis and medication. Only those effects significant at p<.05 are shown, and followed up with post-hoc comparisons using independent-samples t-tests.

### Functional MRI Acquisition and Preprocessing

MRI scanning occurred on a GE 3T Signa scanner (LX [8.3] release). A T1-weighted image was acquired in the same prescription as functional images to facilitate co-registration. Functional images were acquired with a T2*-weighted, reverse spiral acquisition sequence (GRE, Repetition time [TR] = 2000 ms, Echo time [TE] = 30, Flip angle = 90, Field of view = 20, 40 slices, 3 mm thickness, skip = 0, matrix dia. 71 - equivalent to 64×64) sensitive to signal in ventral frontal regions [53]. Data were acquired for 6 minutes while subjects fixated on a crosshair, resulting in 180 volumes (plus 4 initial discarded volumes). Following acquisition of functional data, a high resolution T1 SPGR scan was obtained for anatomic normalization.

Physiologic signals (heart rate and respiration) were removed from the data using RETROICOR [54]. Data were then realigned and slice-time corrected using slicetimer (interpolated with an 8-point sinc kernel multiplied by a Hanning window) (FSL, Analysis Group, FMRIB, Oxford). Functional volumes were coregistered and resliced to 3 mm^3^, normalized to the template MNI152 brain (Montreal Neurological Institute), and smoothed with a 5 mm isotropic Gaussian smoothing kernel using the Statistical Parametric Mapping (SPM) 2 package (Wellcome Institute of Cognitive Neurology, London).

### Functional Connectivity Analysis

Analysis of functional connectivity of LFBFs was carried out with the “conn" toolbox (www.nitrc.org/projects/conn, see ref. [55]. This tool identifies principle components associated with segmented white matter (WM) and cerebrospinal fluid (CSF) images for each individual subject using the “CompCor" method [56]. Critically, this method corrects for positivity biases arising from “noise correlations" related to non-neural sources (such as respiration or cardiac activity) without regressing out the global signal, which has been shown to lead to spurious negative correlations [57]. Timecourses from the top three principle components associated with both WM and CSF were regressed out of whole-brain gray matter activity.

In addition to removing noise correlations present in WM and CSF, the addition of 12 motion regressors (6 realignment parameters and first derivatives) controlled for correlations due to movement. Data were filtered between .01 and .10 Hz. We examined connectivity patterns separately for seven different seed regions-of-interest (ROIs) located in FPN. Given abundant evidence of positive correlations among FPN nodes, the timecourses for these separate seeds are not likely to be completely orthogonal; thus we cannot make strong claims about distinct patterns of connectivity between the different seeds. Nevertheless, given recent evidence identifying dissociable cingulo-opercular and dorsolateral prefrontal and parietal systems within FPN [20], [34], [36], we feel that this approach is more informative than creating a global timecourse compiled from signals averaged across the entire network.

Coordinates (in MNI format) for these seeds were taken from prior studies, which have identified a “core" task-set network that includes bilateral dorsal anterior insula (Brodmann's area [BA] 13, x = −35, y = 14, z = 6; x = 36, y = 16, z = 5) and posterior medial frontal cortex (BA 6/32, x = −1, y = 8, z = 51) [34], and a central executive network consisting of bilateral dorsolateral prefrontal cortex (BA 8/9, x = −45, y = 16, z = −45; x = 45, y = 16, z = −45) and bilateral anterior regions of inferior parietal lobule (BA 40, x = −38, y = −53, z = 45; x = 54, y = −50, z = 50) [36]. Timecourses representing the average within 6 mm-radius spheres located around these coordinates were correlated with all gray matter voxels over the 180 volumes using a Hanning window. Correlation coefficient images between FPN seeds and whole-brain gray matter were z-transformed, with one and two-sample t-tests examining within- and between-group connectivity. Significant clusters were defined using a voxelwise threshold of p<.005 and cluster-level corrected for multiple comparisons using familywise error (FWE) correction at a threshold of p<.05 as implemented in SPM8.

Although not our main focus of analysis, we also sought to investigate within-DMN patterns of connectivity, due to two prior reports of reduced connectivity in OCD [58], [59]. We used seeds located in two regions thought to be core “hubs" of DMN, anterior medial prefrontal cortex (BA 10, x = −6, y = 52, z = −2) and posterior cingulate cortex (BA 31, x = −8, y = −56, z = 26) (coordinates taken from ref. 39). In order to restrict our analysis to connectivity within DMN, within- and between-group t-tests searched for effects in a mask of DMN, which consisted of regions showing positive connectivity with the posterior cingulate (PCC) seed in the uHC group at p<.05 (cluster-level corrected using FWE).

Relationships with symptom severity were examined by extracting connectivity values from regions showing group differences and correlating these with Y-BOCS scores (total score, obsessions subscale, and compulsions subscale) in the OCD group.

Several post-hoc analyses were performed to examine the impact of other variables on results (see Supporting information File S1). Multiple regressions examined the influence of diagnosis (OCD vs. controls) on extracted connectivity values when controlling for medication, generalized anxiety/depression, and education (as the latter two variables differed between the groups, see Table 1). These regressions were repeated for a restricted sample where patients and control groups were matched for head movement [60].

## Results

### Connectivity with fronto-parietal network

There were several regions that showed significant group differences in connectivity with seeds in fronto-parietal network. For all of these regions, there was greater overall connectivity with FPN seeds in OCD patients as compared to controls, due in some cases to patterns of reduced negative correlations in patients and in other cases to patterns of increased positive correlations in patients (see Table 2 for direction of effects in each region).

**Table 2:** Regions where connectivity with fronto-parietal seeds was greater in OCD patients than control subjects.

OCD>CONTROL							
Region	BA	k	x	y	z	Z	Effect in OCD*
***Left anterior insula seed***							
Parahippocampus (R)	27, 36	505±	18	−39	−12	4.4	↑ positivity/↓ negativity
PCC/precuneus/medial	7, 18, 19,	±	6	−78	33	4.4	↑ positivity
occipital (B)	30, 31						
Precuneus/	7, 31	±	21	−60	24	4.0	↓ negativity
medial occipital (B)							
pIPL/posterior	19, 39	77	−36	−78	30	4.0	↑ positivity/↓ negativity
temporal/occipital (L)							
Pre-postcentral (L)	3, 4	69	−30	−30	66	3.9	↑ positivity
DMPFC (B)	8, 9	76	−6	48	36	3.5	↑ positivity/↓ negativity
***Right anterior insula seed***							
PCC/precuneus/	7, 18, 30,	656±	−18	−72	24	4.6	↑ positivity
medial occipital (B)	31						
PCC/precuneus/	7, 29, 31	±	−12	−57	27	4.3	↓ negativity
retrosplinal (B)							
Thalamus/	27	±	−12	−33	3	3.4	↑ positivity
parahippocampus (L)							
Thalamus (R)	N/A	±	15	−27	0	3.5	↑ positivity
Parahippocampus (R)	27	±	15	−36	−3	3.7	↑ positivity
Posterior insula (R)	13	177	30	−21	3	4.1	↑ positivity
DMPFC/aMFC (L)	8, 9, 10	194	−6	30	39	3.8	↑ positivity/↓ negativity
pIPL/posterior	39	134	−48	−63	21	3.9	↑ positivity/↓ negativity
temporal/occipital (L)							
***Left dorsolateral prefrontal seed***							
Medial occipital/	17, 18	426	−9	−75	0	5.1	↑ positivity
cerebellum (B)							
aI/fO (R)	13, 47	75	36	18	−6	4.1	↑ positivity
***Right dorsolateral prefrontal seed***							
PCC/medial occipital (B)	18, 30	218	−9	−75	−3	4.0	↑ positivity
Middle/inferior frontal (R)	45, 46	85	45	39	9	4.2	↑ positivity
Middle/superior frontal (R)	8, 9	89	36	36	42	4.2	↑ positivity
***Left anterior inferior parietal seed***							
Pre-postcentral (L)	3, 4	68	−39	−18	57	3.9	↑ positivity
***Right anterior inferior parietal seed***							
PCC/precuneus (R)	29, 30	101	15	−60	9	3.7	↑ positivity/↓ negativity

Regions shown are corrected for multiple comparisons across the whole-brain at p<.05.

BA = Brodmann's area(s); k = number of voxels; Z = maximum z-score; L = left; R = right; B = bilateral; PCC = posterior cingulate cortex; pIPL = posterior inferior parietal lobule; DMPFC = dorsomedial prefrontal cortex; aMFC = anterior medial prefrontal cortex; aI/fO = anterior insula/frontal operculum; coordinates are in MNI space. ±Coordinates represent subpeaks of cluster.

* Group differences were driven by regions showing positive connectivity in OCD that was reduced or absent in controls (“↑ positivity"), negative connectivity in controls that was reduced or absent in OCD (“↓ negativity"), or positive connectivity in OCD and negative connectivity in controls (“↑ positivity/↓ negativity").

**Figure 1:**
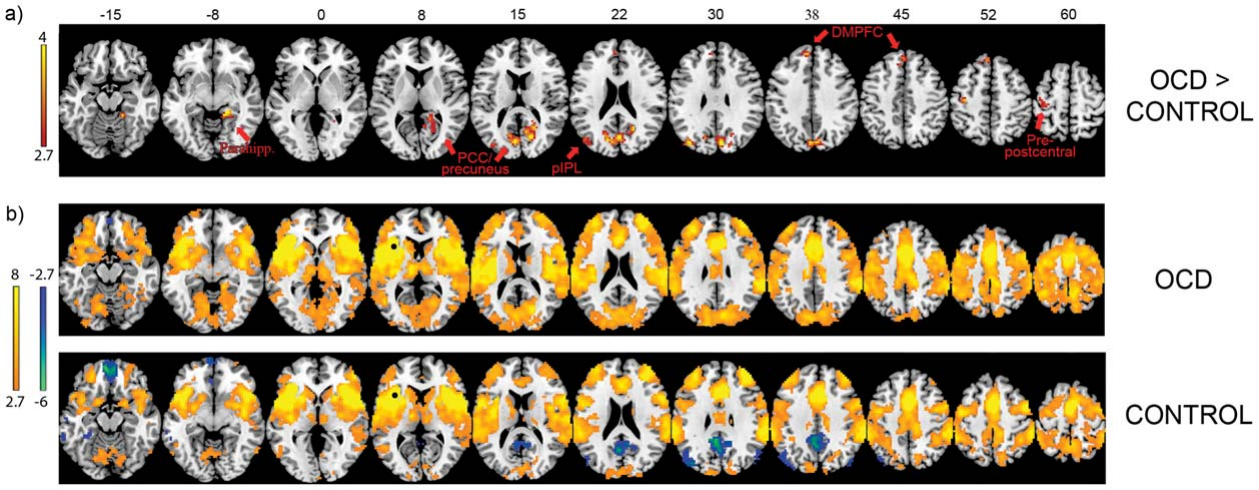
Connectivity with left anterior insula. a) OCD patients exhibited greater connectivity than controls between the left anterior insula seed and default mode network regions including parahippocampus, posterior cingulate cortex (PCC)/precuneus, posterior inferior parietal lobule (pIPL), and dorsomedial prefrontal cortex (DMPFC). Patients also showed greater connectivity with pre-postcentral gyrus. Numbers above images represent z-coordinate (MNI format) of axial slices. Images displayed at p<.005, corrected across whole-brain gray matter at p<.05, b) Connectivity maps with left anterior insula for both groups, displayed at p<.005 with 10 contiguous voxels. Black circles represent location of seed. Color bars represent t-scores.

**Figure 2:**
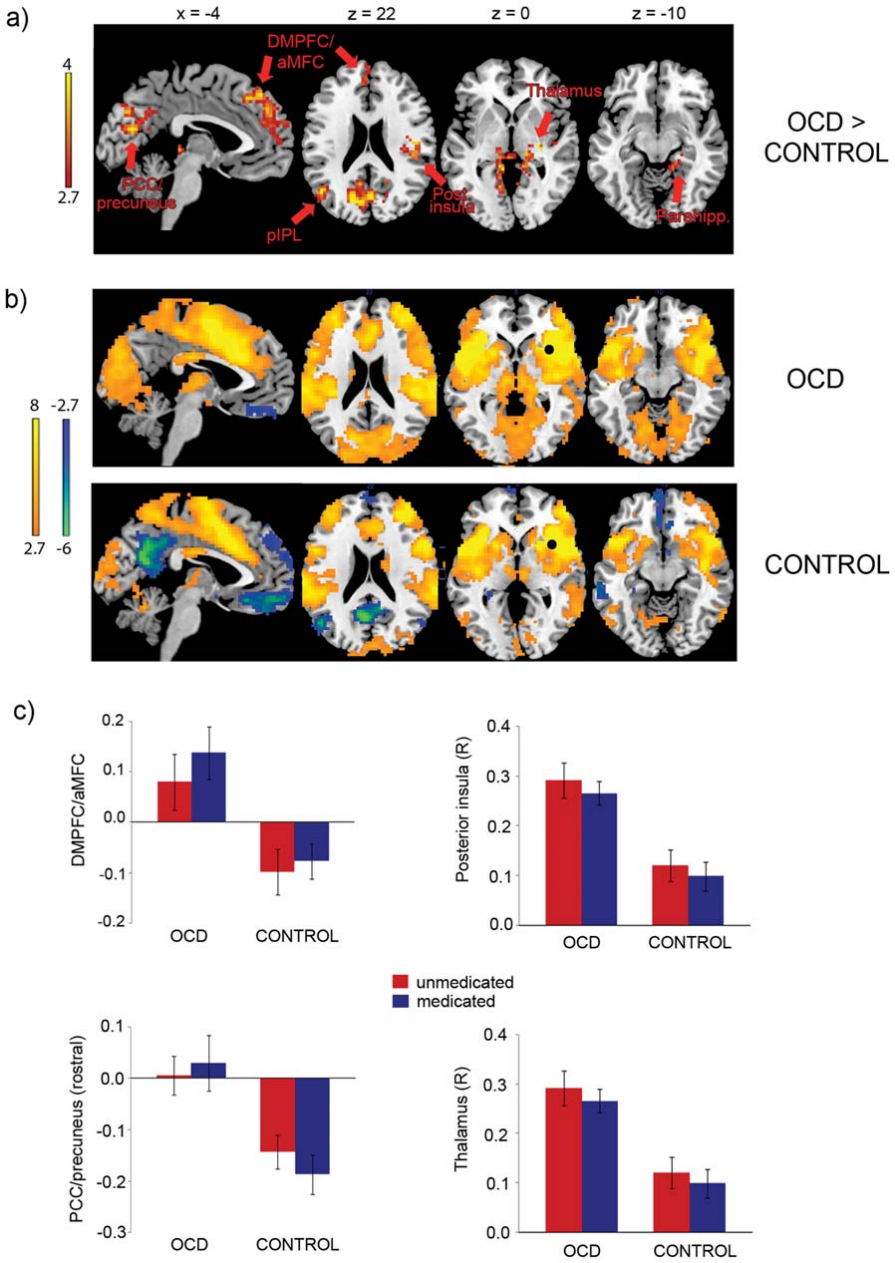
Connectivity with right anterior insula. a) OCD patients exhibited greater connectivity than controls between the right anterior insula seed and default mode network regions including posterior cingulate cortex (PCC)/precuneus, dorsomedial prefrontal cortex (DMPFC)/anterior medial frontal cortex (aMFC), posterior inferior parietal lobule (pIPL), and parahippocampus. Patients also showed greater connectivity with right posterior insula and bilateral thalamus. Images displayed at p<.005, corrected across whole-brain gray matter at p<.05, b) Connectivity maps with right anterior insula seed for both groups, displayed at p<.005 with 10 contiguous voxels. Black circles represent location of seed, c) Extracted connectivity values (y-axes show z-transformed correlation coefficients) in DMPFC/aMFC, rostral PCC/precuneus, right posterior insula, and right thalamus for unmedicated OCD patients (uOCD), medicated OCD patients (mOCD), unmedicated healthy controls (uHC), and medicated patient controls (mPC); uOCD>uHC and mOCD>mPC for all regions shown at p<.05 in multiple regressions controlling for generalized anxiety/depression and education (see File S1 and Table S2). Error bars represent standard error of the mean. Color bars represent t-scores. Coordinates are in MNI format.

For the left anterior insula seed, OCD patients exhibited greater connectivity with several areas of default mode network including PCC/precuneus extending into medial occipital lobe, right parahippocampus, left posterior regions of inferior parietal lobule (pIPL) and adjacent regions of posterior temporal cortex, and dorsomedial prefrontal cortex (DMPFC) (Figure 1). Patients also had greater connectivity between the left anterior insula seed and left pre-postcentral gyrus. Similarly, for the right anterior insula seed, patients exhibited greater connectivity with DMN regions including PCC/precuneus, parahippocampus, left pIPL, and DMPFC extending into anterior MFC (Figure 2). In addition, patients showed greater connectivity between the right anterior insula seed and right posterior insula as well as bilateral thalamus. There were no significant differences in connectivity with the posterior medial frontal seed.

Hyper-connectivity in OCD was also found with dorsolateral and anterior inferior parietal seeds of FPN, although differences with DMN were less robust than with anterior insula seeds. For the left dorsolateral prefrontal (DLPFC) seed, patients had greater connectivity with right anterior insula/frontal operculum – also a part of FPN – as well as ventral occipital lobe/cerebellum. For the right DLPFC seed, patients not only had greater connectivity with the posterior cingulate cortex in the DMN, but also showed increased coupling with right lateral frontal regions of FPN.

Fewer group differences emerged for connectivity with anterior inferior parietal seeds, with the left hemisphere exhibiting more connectivity with left pre-postcentral gyrus and the right hemisphere exhibiting more connectivity with PCC/precuneus for OCD patients as compared to controls.

### Connectivity within default mode network

There were no regions within DMN that exhibited group differences in connectivity with anterior medial frontal (aMFC) or PCC seeds at the current threshold, which was corrected for multiple comparisons. However, as two prior studies have reported altered resting-state connectivity within DMN in OCD [29], [59], we examined connectivity between these seeds and regions of DMN at an uncorrected threshold (p<.005, k = 10). In contrast to results from FPN seeds, where all group differences were associated with less negative and/or more positive connectivity in patients, OCD patients showed less positive connectivity between the aMFC seed and an adjacent region of aMFC (BA 10, k = 12, x = 9, y = 57, z = 9) and with DMPFC (BA 9, k = 21, x = 0, y = 57, z = 27) compared to controls. Patients also showed reduced connectivity between the PCC seed and bilateral aMFC/DMPFC (BA 10, k = 14, x = 9, y = 69, z = 18; k = 10, x = −24, y = 63, z = 12). There were no areas where within-DMN connectivity was greater in OCD patients than controls, even at this lowered threshold.

### Relationships with OC symptoms severity

Connectivity between the right anterior insula seed and right thalamus was significantly related to Y-BOCS scores, with greater severity of symptoms associated with reduced connectivity (total score: r = −0.50, p = 0.005, obsessions subscale: r = −.46, p = .011; compulsions subscale: r = −.45. p = .013). No other connectivity values were related to OC symptom severity.

### Impact of other variables on effects of diagnosis

As is shown in the Supporting information (File S1), group differences persisted after controlling for the influence of medication status, generalized anxiety/depression, education, and head motion on connectivity. Comparisons between unmedicated OCD patients and healthy controls can be seen in Table S2.

## Discussion

Primary results from the current study revealed reduced negative connectivity between the fronto-parietal network and default mode network in OCD. Patients also showed more positive connectivity between fronto-parietal seeds and several areas outside DMN, including somato-motor areas (pre-postcentral gyrus and posterior insula) and other FPN regions (anterior insula/frontal operculum and lateral frontal cortex). However, not all group differences reflected increased connectivity in OCD, as patients showed less positive connectivity than control subjects within DMN itself, although these effects only emerged at lower levels of significance. Negative connectivity among controls was evident despite the fact that global normalization was not used in the analysis [57], and group differences were not due to head motion [60]. Importantly, the factorial design gave us greater ability to control for confounding factors frequently co-occuring in OCD, such as the presence of medication and depression, and post-hoc multiple regression analyses indicated that these variables did explain group differences. Overall, these data suggest that intrinsic functional relationships between large-scale brain systems are altered in OCD, specifically pointing to a disruption of the competitive interactions typically found at rest between fronto-parietal and default mode networks.

Research on DMN has garnered much interest since the initial observation of reduced blood flow within several anatomically-widespread brain regions during cognitive tasks versus passive viewing [38]. Subsequent studies have associated this network with a core set of regions including PCC/precuneus, ventral and dorsal medial frontal cortex, posterior inferior parietal cortex, and medial and lateral temporal cortex [40]. Activation in these regions not only decreases during externally-directed attention, but also increases when attention is directed inward, such as when subjects think about their own personal qualities and take the perspective of others (theory of mind/mentalizing), remember personal episodes from their past, imagine future events, and construct scenes [37], [39], [40], [61]. Experiments investigating the relationship between fronto-parietal network regions involved in executive/control processes and DMN indicate that efficient processing of external stimuli requires the suspension of introspectively-oriented cognition [44], [45], and that this inverse pattern of activity appears even when the brain is at rest [18], [35], [46].

Recent evidence has identified dissociable sub-networks within FPN, with dorsal regions of anterior insula and medial frontal cortex forming a “core" network involved in implementing and maintaining attention to external task demands and detecting salient events [20], and lateral frontal and parietal regions performing more “executive" functions such as working memory, planning, and cognitive control [20], [36]. Right insula in particular may function as a “switch" between modes of processing, triggering the activation of executive regions and suppression of default mode regions when salient external events are detected [36], [62]. While anterior insula may respond somewhat generally to external task demands, it does appear to be particularly sensitive to stimuli signaling potential risk [63]; as such, this area may be important for switching attention away from an internal focus toward the external environment after detecting potentially harmful situations. Though speculative, the current findings of attenuated negative connectivity between anterior insula and DMN suggest a potential neural basis for the difficulty OCD patients may have in efforts to disengage from internal mental processes in order to respond more appropriately to salient external information related to potential risk (such as that informing them that dreaded events have not or will not occur). These findings extend work identifying greater connectivity between anterior insula and VMPFC during task [9] to include alterations in connectivity with several regions of DMN at rest, and raise the possibility that abnormal intrinsic connectivity contributes to the DMN hyperactivity found in OCD during cognitive tasks [8], [9], [58], [64].

Connectivity differences with FPN occurred across a number of DMN regions, including PCC, pIPL, DMPFC/aMFC, and parahippocampus. Despite the consistent activation of these brain regions across several different tasks involving internally-directed cognition [61], there appears to be some measure of dissociation between different nodes within DMN, particularly between anterior medial frontal and posterior parietal/temporal regions [19], [65], [66]. Although the current study was not aimed at distinguishing among different DMN subsystems, the findings of aberrant connectivity between FPN and several DMN regions suggests that altered resting-state connectivity in OCD is not specific to a particular DMN subsystem.

OCD patients also showed greater positive interactions between FPN seeds and several areas outside of DMN, including pre-postcentral gyrus and posterior insula, which are part of a somatosensory/interoceptive and motor network [67]. Increased connectivity between fronto-parietal regions and somato-motor network could contribute to obsessive-compulsive symptoms involving sensitivity to physical sensations, particularly those related to disgust or internal urges, although such interpretations need to be tested directly. The finding of hyper-connectivity between anterior insula and thalamus is also of interest given the importance of the fronto-striatal-thalamic circuitry in OCD [2]–[4]. Whereas positive connectivity between these regions was greater in the patient group overall, it was inversely related to OC symptom severity, suggesting a compensatory mechanism because patients with lesser severity of symptoms showed the greatest difference from controls. However, this finding should be interpreted with caution as we did not correct for the number of tests that were performed to examine correlations with symptom severity.

Although the general absence of correlations with Y-BOCS scores might seem surprising, this may indicate that group differences reflect stable biomarkers of OCD not sensitive to symptom severity differences, similar to the mechanism suggested for the error-related negativity [68]. The current analysis focused on identifying connectivity patterns where OCD patients differed from both healthy controls and remitted depressed subjects (see Figure 2c and Table S2). However, the current data cannot address whether similar disturbances would be found in patients with active major depression, or whether remitted depressed patients would show differences in connectivity compared to healthy controls in brain regions other than those explored in the current analysis.

Unlike group differences found with FPN seeds, OCD patients showed less positive connectivity than controls subjects within DMN, a finding that was not due to differential motion. Although this effect was found only at a lowered threshold, it is consistent with two prior reports [58], [59], and is intriguing given the finding that negative affect reduces connectivity within DMN [69]. These results suggest that OCD may be characterized by a complex pattern of hyper- and hypo-connectivity among large-scale networks.

To our knowledge, this is the first report of altered intrinsic connectivity between distributed regions of fronto-parietal and default mode networks in OCD. However, there are several limitations of the current study, many of which could be addressed by future research. Given that results were not corrected for multiple seed comparisons, replications using a larger sample size are necessary. In addition, OCD and controls groups were not matched on generalized depression/anxiety and education levels. Although post-hoc inclusion of these factors in multiple regressions indicated that these effects were not driving the reported group differences in connectivity, future studies would benefit from investigating the effects of these variables on brain connectivity. Of particular interest for the study of psychopathology, hyperactivity in DMN has been identified in other psychiatric disorders (for a review, see [70]), and the current investigation cannot address whether the findings are specific to OCD. It is possible that changes in DMN across a range of dysfunction could be due to abnormal relationships between FPN and DMN, which would be consistent with the emerging recognition that overlapping physiological mechanisms are found among comorbid disorders [71]. Many anxiety disorders [72], including OCD [8], [9], [73], [74], are characterized by increased activation of anterior insula; as such, the hyper-connectivity between insula and DMN revealed by the current study may be more related to an anxiety phenotype than other psychopathologies, although direct comparisons between disorders will be needed to test this hypothesis. In addition, although we did not have enough subjects to examine different symptom dimensions in OCD, it is likely that some DMN-based cognitive processes (e.g., scene construction and imagination) are more directly related to certain OCD subtypes (e.g., those involving intrusive imagery) than others (e.g., those with symmetry/ordering concerns). Future work should be aimed at obtaining a large and diverse cohort of patients to determine whether these alterations in rs-fcMRI differ based on symptom dimension. Finally, although interpretations regarding the functional significance of resting-state interactions rely on a large body of literature in cognitive neuroscience, it will be necessary to directly test the relationship between rs-fcMRI and behavior as well as task-evoked activity in order to elucidate the cognitive processes associated with these abnormal interactions. In particular, assessment of subjects' thoughts or feelings during rest will improve the ability to link resting-state connectivity to behavior. Despite these issues, the current results highlight the importance of inter-regional interactions between large-scale networks in OCD, revealing alterations of connectivity that may provide promising leads for the development of novel treatments using behavioral training or neuromodulation to target specific patterns of dysfunctional connectivity. Future work will be aimed at replicating and extending the current findings in order to determine the generality of the effect and its behavioral significance.

## Supporting Information

File S1 **Details of Methods and** **Results** **.**
(DOCX)

Table S1 **Medications taken by OCD patients (mOCD) and medicated control subjects (mPC).** SSRIs = selective-serotonin reuptake inhibitors; SNRIs = serotonin-norepinephrine reuptake inhibitors; TCAs = tricycle antidepressants. All subjects except 1 mOCD patient were taking a serotonin reuptake inhibitor (SSRI or SNRI). Two mOCD and 3 mPC subjects were taking more than one medication (not including benzodiazepines, which were taken as needed and omitted on the day of testing).
(DOCX)

Table S2 **Significance of diagnosis in predicting connectivity separately for unmedicated and medicated participants.** Results obtained from multiple regressions also including generalized anxiety/depression and education as predictors. PCC = posterior cingulate cortex; pIPL = posterior inferior parietal lobule; DMPFC = dorsomedial prefrontal cortex; aMFC = anterior medial frontal cortex; aI/fO = anterior insula/frontal operculum.
(DOCX)
