# Supplementary material for: Resting-State Functional Connectivity between Fronto-Parietal and Default Mode Networks in Obsessive-Compulsive Disorder
Source: PLoS One. 2012 May 3;7(5):e36356. doi: 10.1371/journal.pone.0036356 (PMC3343054; doi:10.1371/journal.pone.0036356)
Supplement: File S1 — Details of Methods and Results . (DOCX) [file pone.0036356.s001.docx]

Materials and Methods

Post-hoc multiple regressions examining the impact of other variables on connectivity

*Effects of medication, generalized anxiety/depression, and education*

As our sample contained both medicated and unmedicated subjects, and because OCD patients showed significantly greater levels of generalized anxiety and depression than controls (as would be expected, see Table 1) as well as fewer years of education, we sought to determine whether these factors were influencing group differences in connectivity. Thus, connectivity values (z-transformed correlation coefficients) from regions that differed significantly between the groups were submitted to multiple regressions that included diagnosis (OCD, controls), medication (unmedicated, medicated), a combined HAM-A/HAM-D score (average of the two measures for each subject, due to high collinearity between individual scores), and education (in years) to determine whether diagnosis would remain significant when controlling for these factors. For regions showing homogenous patterns of connectivity within each group (e.g., where the entire area of group difference was driven by positive correlations in OCD that were absent in controls), connectivity values used in multiple regressions were averaged across the entire cluster. For larger regions (>500 voxels) that showed heterogeneous patterns of connectivity within each group (those clusters marked with “±” in Table 2 in the main manuscript, where e.g., caudal portions of the region of group difference showed positive correlations in OCD that were absent in controls, while rostral portions showed negative correlations in controls that were absent in OCD), regression analyses used connectivity values averaged within 6 mm-radius spheres located around the peak and subpeak(s) of the cluster.

*Effect of movement*

Due to evidence highlighting the impact of in-scanner head motion on functional connectivity results [1], six realignment parameters (x, y, z, roll, pitch, yaw) were inspected to ensure that movement did not exceed 3 mm translation or 1 degree of rotation for all participants. Despite these criteria, on average OCD patients rotated significantly more in the pitch direction than control subjects (F (1, 58) = 4.1, p = .047), although none of the other 5 parameters were significantly different between the groups. In order to determine whether this group difference could be impacting results, we repeated the above-described multiple regressions after matching the OCD and control groups for movement (an approach recommended by ref. 1) by excluding 11 participants (7 OCD and 5 control) who exhibited average movement (across the timecourse) in any of the 6 parameters investigated that was 2 standard deviations from the mean of all subjects.

Results

Effects of medication, generalized anxiety/depression, and education on diagnosis predictor

Analyses revealed that all the reported differences between OCD patients and controls remained significant after controlling for effects of medication status, generalized anxiety/depression, and education. This indicates that, despite group differences for these variables, they do not significantly explain the connectivity findings observed. Table S2 shows results from multiple regressions examining connectivity in unmedicated and medicated participants separately, indicating that the majority of differences between patients and controls remained significant despite the reduction of sample sizes by half.

Effect of movement on diagnosis predictor

None of the 6 movement parameter values differed significantly between patients and controls after excluding participants showing movement over 2 SD from the mean. Critically, all effects of diagnosis reported remained significant after removing these subjects, with the exception of right aIPL connectivity with PCC/precuneus and PCC connectivity with left aMFC, where effects of diagnosis were reduced to trend level significance (p = .062 and .081, respectively). This indicates that differential movement between the groups was not responsible for current findings.

References

1. Van Dijk KRA, Sabuncu MR, Buckner RL (2012) The influence of head motion on intrinsic functional connectivity MRI. NeuroImage 59: 431-438.
