# Supplementary material for: Resting-State Functional Connectivity between Fronto-Parietal and Default Mode Networks in Obsessive-Compulsive Disorder
Source: PLoS One. 2012 May 3;7(5):e36356. doi: 10.1371/journal.pone.0036356 (PMC3343054; doi:10.1371/journal.pone.0036356)
Supplement: Table S1 — Medications taken by OCD patients (mOCD) and medicated control subjects (mPC). SSRIs = selective-serotonin reuptake inhibitors; SNRIs = serotonin-norepinephrine reuptake inhibitors; TCAs = tricycle antidepressants. All subjects except 1 mOCD patient were taking a serotonin reuptake inhibitor (SSRI or SNRI). Two mOCD and 3 mPC subjects were taking more than one medication (not including benzodiazepines, which were taken as needed and omitted on the day of testing). (DOCX) [file pone.0036356.s002.docx]

**Table S1. Medications taken by OCD patients (mOCD) and medicated control subjects (mPC).**

| **Medications** | **N in mOCD group** | **Average dosage (mg)** | **N in mPC group** | **Average dosage (mg)** |
| --- | --- | --- | --- | --- |
| **SSRIs/SNRIs** |  |  |  |  |
| Citalopram | 2 | 40 | 1 | 40 |
| Escitalopram | 3 | 36.6 | 7 | 16.43 |
| Fluoxetine | 4 | 47.5 | 1 | 30 |
| Fluvoxamine | 1 | 250 |  |  |
| Paroxetine | 1 | 60 |  |  |
| Sertraline |  |  | 1 | 100 |
| Venlafaxine | 1 | 187.5 | 5 | 270 |
| **TCAs** |  |  |  |  |
| Clomipromine | 1 | 100 |  |  |
| **Benzodiazepines** |  |  |  |  |
| Alprazolam | 3 | 0.75 |  |  |
| Clonazepam | 3 | 0.5 | 1 | 0.375 |
| Lorazepam | 1 | 0.5 | 3 | 0.67 |
| **Other** |  |  |  |  |
| Buproprion | 1 | 300 | 2 | 300 |
| Buspirone |  |  | 1 | 20 |
| Gabapentin | 1 | 600 |  |  |
| Methylphenidate |  |  | 1 | 54 |
| Trazadone | 1 | 100 |  |  |

SSRIs = selective-serotonin reuptake inhibitors; SNRIs = serotonin-norepinephrine reuptake inhibitors; TCAs = tricycle antidepressants. All subjects except 1 mOCD patient were taking a serotonin reuptake inhibitor (SSRI or SNRI). Two mOCD and 3 mPC subjects were taking more than one medication (not including benzodiazepines, which were taken as needed and omitted on the day of testing).
