# Supplementary material for: Resting-State Functional Connectivity between Fronto-Parietal and Default Mode Networks in Obsessive-Compulsive Disorder
Source: PLoS One. 2012 May 3;7(5):e36356. doi: 10.1371/journal.pone.0036356 (PMC3343054; doi:10.1371/journal.pone.0036356)
Supplement: Table S2 — Significance of diagnosis in predicting connectivity separately for unmedicated and medicated participants. Results obtained from multiple regressions also including generalized anxiety/depression and education as predictors. PCC = posterior cingulate cortex; pIPL = posterior inferior parietal lobule; DMPFC = dorsomedial prefrontal cortex; aMFC = anterior medial frontal cortex; aI/fO = anterior insula/frontal operculum. (DOCX) [file pone.0036356.s003.docx]

**Table S2.** **Significance of diagnosis in predicting connectivity separately for unmedicated and medicated participants.**

| **Region** | **uOCD > uHC** | **mOCD > mPC** |
| --- | --- | --- |
| **Fronto-parietal network connectivity** | | |
| ***Left anterior insula seed*** | | |
| Parahippocampus (R) | .046 | .005 |
| PCC/precuneus/medial | .026 | .07 |
| occipital (B) |  |  |
| Precuneus/ | .082 | .022 |
| medial occipital (B) |  |  |
| pIPL/posterior | .033 | .006 |
| temporal/occipital (L) |  |  |
| Pre-postcentral (L) | .451 | .003 |
| DMPFC (B) | .024 | .009 |
| ***Right anterior insula seed*** | | |
| PCC/precuneus/ | .163 | .013 |
| medial occipital (B) |  |  |
| PCC/precuneus/ | .010 | .003 |
| retrosplinal (B) |  |  |
| Thalamus/ | .014 | .017 |
| parahippocampus (L) |  |  |
| Thalamus (R) | .030 | .004 |
| Parahippocampus (R) | .192 | .003 |
| Posterior insula (R) | .008 | < .001 |
| DMPFC/aMFC (L) | .028 | .006 |
| pIPL/posterior | .032 | < .001 |
| temporal/occipital (L) |  |  |
| ***Left dorsolateral prefrontal seed*** | |  |
| Medial occipital/ | .083 | < .001 |
| cerebellum (B) |  |  |
| aI/fO (R) | .146 | < .001 |
| ***Right dorsolateral prefrontal seed*** | | |
| PCC/medial occipital (B) | .003 | .003 |
| Middle/inferior frontal (R) | .251 | .009 |
| Middle/superior frontal (R) | .099 | .001 |
| ***Left anterior inferior parietal seed*** | | |
| Pre-postcentral (L) | .031 | .117 |
| ***Right anterior inferior parietal seed*** | | |
| PCC/precuneus (R) | .159 | .017 |
| **Within-default mode network connectivity** | | |
| ***Anterior medial frontal seed*** | | |
| aMFC (R) | .030 | .033 |
| DMPFC (B) | .045 | .005 |
| ***Posterior cingulate seed*** | | |
| aMFC/DMPFC (R) | .105 | < .001 |
| aMFC (L) | .084 | .172 |

Results obtained from multiple regressions also including generalized anxiety/depression and education as predictors. PCC = posterior cingulate cortex; pIPL = posterior inferior parietal lobule; DMPFC= dorsomedial prefrontal cortex; aMFC = anterior medial frontal cortex; aI/fO = anterior insula/frontal operculum.
